# Small graft size and hepatocellular carcinoma outcomes in living donor liver transplantation: a retrospective multicentric cohort study

**DOI:** 10.1097/JS9.0000000000001532

**Published:** 2024-05-03

**Authors:** Deok-Gie Kim, Shin Hwang, Kwang-Woong Lee, Jong Man Kim, Young Kyoung You, Donglak Choi, Je Ho Ryu, Bong-Wan Kim, Dong-Sik Kim, Jai Young Cho, Yang Won Nah, Man ki Ju, Tae-Seok Kim, Jae Geun Lee, Myoung Soo Kim, Alessandro Parente, Ki-Hun Kim, Andrea Schlegel, Soo Jin Na Choi, Dong Jin Joo

**Affiliations:** a Department of Surgery, The Research Institute for Transplantation, Yonsei University College of Medicine; b Department of Surgery, College of Medicine University of Ulsan, Asan Medical Center; c Department of Surgery, Seoul National University College of Medicine; d Department of Surgery, Samsung Medical Center, Sungkyunkwan University School of Medicine; e Department of Surgery, College of Medicine, The Catholic University of Korea; f Division of HBP Surgery and Liver Transplantation, Department of Surgery, University College of Medicine; g Departmentof Surgery, Gangnam Severance Hospital, Yonsei University College of Medicine, Seoul; h Department of Hepato-Biliary-Pancreatic Surgery, Ajou University School of Medicine, Suwon; i Department of Surgery, Catholic University of Daegu; j Department of Surgery, Dongsan Medical Center, Keimyung University School of Medicine, Daegu; k Department of Surgery, Ulsan University Hospital, University of Ulsan College of Medicine, Ulsan; l Department of Surgery, Pusan National University Yangsan Hospital, Pusan National University School of Medicine, Pusan; m Department of Surgery, Seoul National University Bundang Hospital, Seoul National University College of Medicine, Seongnam; n Department of Surgery, Chonnam National University Medical School, Chonnam National University Hospital, Gwangju, South Korea; o Transplantation Center, Department of Surgery, Digestive Disease Institute, Cleveland Clinic, Ohio, USA

**Keywords:** graft-recipient weight ratio, hepatocellular carcinoma, KOTRY, living donor liver transplantation, recurrence

## Abstract

**Introduction:**

This study examined associations between the graft-to-recipient weight ratio (GRWR) for adult-to-adult living donor liver transplantation (LDLT) and hepatocellular carcinoma (HCC) outcomes.

**Materials and Methods:**

Data from patients in the Korean Organ Transplantation Registry who underwent LDLT for HCC from 2014 to 2021 were retrospectively reviewed. Patients were categorized using the cutoff GRWR for HCC recurrence determined by an adjusted cubic spline (GRWR <0.7% vs. GRWR ≥0.7%). Recurrence-free survival (RFS) and HCC recurrence were analyzed in the entire and a 1:5 propensity-matched cohort.

**Results:**

The eligible cohort consisted of 2005 LDLT recipients [GRWR <0.7 (*n*=59) vs. GRWR ≥0.7 (*n*=1946)]. In the entire cohort, 5-year RFS was significantly lower in the GRWR <0.7 than in the GRWR ≥0.7 group (66.7% vs. 76.7%, *P*=0.019), although HCC recurrence was not different between groups (77.1% vs. 80.7%, *P*=0.234). This trend was similar in the matched cohort (*P*=0.014 for RFS and *P*=0.096 for HCC recurrence). In multivariable analyses, GRWR <0.7 was an independent risk factor for RFS [adjusted hazard ratio (aHR) 1.89, *P*=0.012], but the result was marginal for HCC recurrence (aHR 1.61, *P*=0.066). In the pretransplant tumor burden subgroup analysis, GRWR <0.7 was a significant risk factor for both RFS and HCC recurrence only for tumors exceeding the Milan criteria (aHR 3.10, *P*<0.001 for RFS; aHR 2.92, *P*=0.003 for HCC recurrence) or with MoRAL scores in the fourth quartile (aHR 3.33, *P*<0.001 for RFS; aHR 2.61, *P*=0.019 for HCC recurrence).

**Conclusions:**

A GRWR <0.7 potentially leads to lower RFS and higher HCC recurrence after LDLT when the pretransplant tumor burden is high.

## Introduction

Highlights

- Evidence for the oncologic impact of small graft size in living donor liver transplantation is lacking.
- This multicentric study demonstrated graft-recipient weight ratio <0.7 showed higher recurrence of hepatocellular carcinoma, especially when the tumor burden was high [adjusted hazard ratio (aHR) 3.10 for above Milan cancer and aHR 3.33 for fourth quartile of MoRAL].

Liver transplantation (LT) is an accepted curative treatment for patients affected by hepatocellular carcinoma (HCC). In countries with a limited deceased donor pool, living donor liver transplantation (LDLT) can provide excellent outcomes for HCC patients. Some reports have even shown survival advantages for LDLT over deceased donation when considering patients with HCC^1–4^. However, other studies have described unfavorable oncological outcomes after LDLT for HCC^5–8^. These studies were based on mechanisms such as parenchymal regeneration of the graft and ischemia-reperfusion injury in small-sized livers, which can contribute to tumor growth after LDLT^9–11^.

A graft-to-recipient weight ratio (GRWR) of 0.8 has been traditionally suggested as the lower limit for safe LDLT^12^. Although several single-center studies reported feasible LDLT outcomes using smaller grafts^13–15^, our recent multicentric data revealed that LDLT with grafts with a GRWR <0.8 resulted in decreased graft survival, especially in the presence of multiple risk factors^16^. The impact of graft size on the HCC outcome in LDLT has been evaluated in a limited number of studies. Lee *et al*.^17^ reported that LDLT with a GRWR <0.8 led to lower recurrence-free survival (RFS) compared to a GRWR ≥0.8 when the tumor exceeded the Milan criteria, although they did not present a difference in time to recurrence. A recent meta-analysis supported these findings, explaining that small-for-size syndrome could be a contributor to HCC recurrence, especially in patients with high tumor burden^18^. However, this subject needs more evidence from multicentric data.

This study aimed to assess the association between small graft size and HCC outcomes in patients who underwent LDLT using data from a large nationwide registry.

## Materials and methods

### Study population

We conducted a multicenter, retrospective cohort analysis using data from 2535 patients in the Korean Organ Transplantation Registry (KOTRY) who received LDLT for HCC between May 2014 and December 2021. The KOTRY is a prospectively maintained database for which details were previously reported^19^. We excluded patients who died or received retransplantation too early (≤30 d) to be investigated for the impact of graft size on HCC outcome (*n*=53). Patients with the following were also excluded: age <18 years (*n*=3), combined cholangiocellular cancer (*n*=119), grafts from dual living donors (*n*=26), retransplantation (*n*=6), and missing data (*n*=323). The remaining 2005 LDLT recipients were included in the analyses (Supplementary Fig. S1, Supplemental Digital Content 2, http://links.lww.com/JS9/C491).

All study procedures were conducted in accordance with the Declaration of Helsinki, as revised in 2013. The institutional review board approved the study (4-2023-1550), and patient consent for this study was waived because of its retrospective design. This retrospective study has been reported in line with the STROCSS criteria^20^ (Supplemental Digital Content 1, http://links.lww.com/JS9/C490).

### Data collection and outcomes

All relevant data regarding donors, recipients, and LDLT surgery were obtained from the KOTRY database. The underlying liver disease was classified as hepatitis B, C, or non-B/non-C. Graft types were classified as either right lobe or other than-right lobe (mostly left lobe grafts). Based on the pathologic examination conducted during donor surgery, graft steatosis was classified into two categories: >10% and ≤10%. Exact information about explant tumor pathology and tumor markers [alpha-fetoprotein (AFP) and protein induced by vitamin K absence or antagonist-II (PIVKA II)] measured at LDLT was obtained to adjust the tumor burden. Data regarding pretransplant treatment for HCC were collected, including prior hepatectomy and pretransplant locoregional and systemic treatment. The primary outcomes were RFS and HCC recurrence (time to recurrence). Patients were monitored until death, retransplantation, 31 December 2022, or 5 years following transplantation, whichever came first.

### Cutoff for categorization of the GRWR

**Figure 1:**
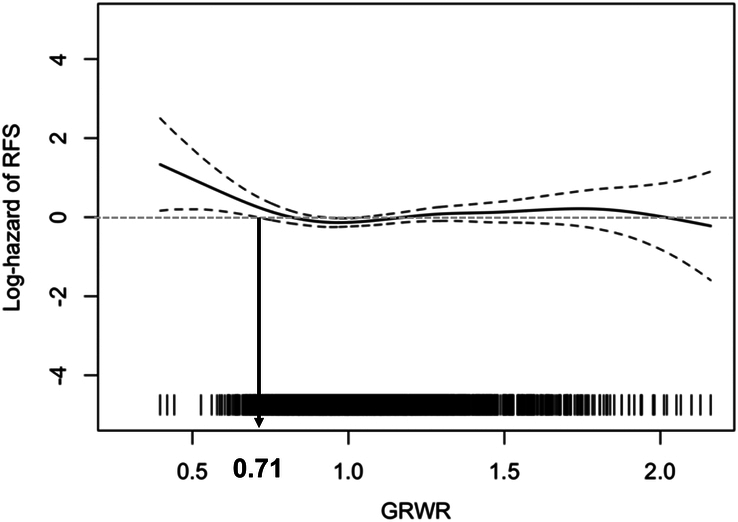
Adjusted spline curve for the hazard of recurrence-free survival according to GRWR. The cutoff for GRWR was determined at which hazard of RFS became significant on the spline curve. Adjusted covariates were the same as those in multivariable Cox. GRWR, graft-recipient weight ratio; RFS, recurrence-free survival.

The graft weight was measured immediately before graft implantation, and the following formula was used to calculate the GRWR [graft weight (g)÷recipient weight (g)]×100. The correlation between GRWR and the hazard of RFS was determined using a smoothing spline curve adjusted using all independent risk factors for RFS (Fig. 1)^21^. The hazard for RFS started to become significant as the GRWR decreased below 0.71. Therefore, for the convenience of analyses and further clinical utilization, a GRWR <0.7 was selected as the cutoff for HCC outcomes.

### Statistical methods

Depending on the type of variable, the data are shown as either numbers (percentages) or medians (IQR). The Mann–Whitney *U* test or the *χ* ^2^ test was used to compare continuous and categorical variables as appropriate. Graft survival was compared using Kaplan–Meier curves and the log-rank test. These analyses were performed both in the entire population and the propensity score (PS)-matched population. The GRWR <0.7 and GRWR ≥0.7 groups were matched in a 1:5 ratio using the nearest neighbor method with a 0.1 caliper. All baseline variables were used to generate PSs, and the matching balance was deemed satisfactory if the standardized mean differences across groups were less than 0.1^22^. If there was no suitable match, patients were eliminated from both groups.

Multivariable Cox regression was performed for HCC outcomes in the entire population including covariates with *P* values <0.1 in the univariable analysis of the model. Non-HCC death was considered a competing risk in the risk analysis for HCC recurrence, which employed competing risk regression utilizing the Fine and Gray approach^23^. Subgroup analyses were performed according to tumor burden based on the Milan criteria^24^, which reflect the tumor number and size, and the MoRAL score, which was developed according to the pretransplant AFP and PIVKA II levels, specifically for the LDLT cohort^25^. Subgroups with high tumor burden included those who exceeded the Milan criteria or had a MoRAL score in the fourth quartile, and the risk of a GRWR <0.7 was evaluated in each subgroup after adjusting for the same covariates as those used for the entire population. All analyses were performed using the R statistical package version 4.3.0 for MacOS (http://cran.r-project.org/), with the threshold for significance set at *P*<0.05.

## Results

### Distribution of the GRWR

The GRWR varied from 0.4 to 2.16 (Supplementary Fig. S2, Supplemental Digital Content 2, http://links.lww.com/JS9/C491), with a median of 1.04 (IQR, 0.90–1.21), among the eligible adult LDLT patients with HCC. Ten patients (0.5%) had GRWR values <0.6; 49 patients (2.4%) had GRWR values of 0.6–0.7; 163 patients (8.1%) had GRWR values of 0.7–0.8; 637 patients (31.8%) had GRWR values of 0.8–1.0; and 1146 patients (57.2%) had GRWR values ≥1.0. As determined by an adjusted cubic spline (see the ‘Methods’ section, Fig. 1), patients were categorized into two groups, the GRWR <0.7 group (*n*=59) and the GRWR ≥0.7 group (*n*=1946).

### Baseline characteristics

As shown in Table 1, the GRWR <0.7 group was younger (56 [51–59] years vs. 57 [52–62] years, *P*=0.004) and had a higher body mass index (BMI) than the GRWR ≥0.7 group (26.2 [24.7–28.5] kg/m^2^ vs. 24.3 [22.2–26.4] kg/m^2^, *P*<0.001). The ABO incompatibility (25.4% vs. 24.3%, *P*=0.958), donor age (31 [24–43] vs. 30 [24–38], *P*=0.282), and Model for End-Stage Liver Disease score (11 [8–15] vs. 10 [8–14], *P*=0.179) were comparable between the groups. The GRWR <0.7 group had a lower donor BMI (21.6 [19.4–23.3] kg/m^2^ vs. 23.6 [21.6–25.6] kg/m^2^, *P*<0.001) and a lower frequency of male donors (40.7% vs. 64.3%, *P*<0.001) than the GRWR ≥0.7 group. Graft steatosis >10% was similar (8.5% vs. 12.6%, *P*=0.599) between the groups, although grafts other than the right lobe were more common in the GRWR <0.7 group than in the GRWR ≥0.7 group (14.4% vs. 3.4%, *P*<0.001).

**Table 1:** Baseline characteristics before and after matching.

	Before matching			After matching		
Variables	GRWR <0.7 (*n*=59)	GRWR ≥0.7 (*n*=1946)	*P*	GRWR <0.7 (*n*=48)	GRWR ≥0.7 (*n*=198)	SMD^a^
Age	56 (51–59)	57 (52–62)	0.044	56 (51–59)	57 (52–61)	0.02
Sex, male	55 (93.2)	1617 (83.1)	0.060	45 (93.8)	185 (93.4)	0.03
BMI, kg/m^2^	26.2 (24.7–28.5)	24.3 (22.2–26.4)	<0.001	25.9 (24.3–28.4)	25.4 (23.6–27.9)	0.09
Underlying disease			0.684			0.04
Hepatitis B	45 (76.3)	1484 (76.3)		37 (77.1)	158 (79.8)	
Hepatitis C	6 (10.2)	147 (7.6)		4 (8.3)	17 (8.6)	
Non-B/Non-C	8 (13.6)	315 (16.2)		7 (14.6)	23 (11.6)	
Hypertension	13 (22.0)	494 (25.4)	0.666	11 (22.9)	43 (21.7)	0.02
Diabetes mellitus	12 (20.3)	589 (30.3)	0.135	10 (20.8)	45 (22.7)	0.02
MELD	11 (8–15)	10 (8–14)	0.179	10 (8–14)	11 (8–14)	0.04
ABO incompatibility	15 (25.4)	472 (24.3)	0.958	12 (25.0)	59 (29.8)	0.09
Living unrelated donor	13 (22.0)	269 (13.8)	0.110	12 (25.0)	41 (20.7)	<0.01
Donor age	31 (24–43)	30 (24–38)	0.282	31 (24–43)	33 (25–44)	0.06
Donor sex, male	24 (40.7)	1252 (64.3)	<0.001	22 (45.8)	88 (44.4)	0.06
Donor BMI, kg/m^2^	21.6 (19.4–23.3)	23.6 (21.6–25.6)	<0.001	21.6 (19.5–23.1)	21.2 (19.8–23.7)	0.08
Graft steatosis ≧10%	5 (8.5)	225 (11.6)	0.599	5 (10.4)	20 (10.1)	0.06
Other than the right graft	10 (16.9)	72 (3.7)	<0.001	6 (12.5)	18 (9.1)	0.05
AFP at LT, ng/ml	5.2 (2.8–28.6)	6.4 (3.2–23.9)	0.707	4.6 (2.8–24.7)	7.2 (3.2–31.5)	0.09
PIVKA II at LT, mAU/ml	31 (19–60)	29 (19–73)	0.625	30 (19–57)	28 (19–68)	0.04
Pretransplant LRT	48 (81.4)	1546 (79.4)	0.846	37 (77.1)	158 (79.8)	0.10
Pretransplant systemic treatment	1 (1.7)	53 (2.7)	0.942	1 (2.1)	4 (2.0)	0.03
Prior liver resection	5 (8.5)	285 (14.6)	0.254	4 (8.3)	19 (9.6)	0.06
Milan criteria, above	17 (28.8)	618 (31.8)	0.736	12 (25.0)	74 (37.4)	0.05
Viable tumor number	2.0 (1.0–2.0)	1.0 (1.0–3.0)	0.283	1.0 (1.0–2.0)	1.0 (1.0–3.0)	0.06
Maximum tumor size, mm	1.9 (1.4–2.8)	1.9 (1.0–3.1)	0.463	1.9 (1.4–2.8)	1.9 (0.9–3.0)	0.07
Sum of tumor size, mm	2.5 (1.8–4.1)	2.3 (1.0–4.2)	0.306	2.4 (1.8–3.9)	2.0 (1.0–3.9)	0.03
Microvascular invasion	17 (28.8)	494 (25.4)	0.657	15 (31.2)	49 (24.7)	0.05
Satellite nodule	20 (33.9)	417 (21.4)	0.034	14 (29.2)	60 (30.3)	0.05
Poor differentiation	11 (18.6)	396 (20.3)	0.876	10 (20.8)	32 (16.2)	0.05
PVTT	2 (3.4)	52 (2.7)	1.000	1 (2.1)	8 (4.0)	0.08

a SMDs were presented as absolute values.

AFP, alpha-fetoprotein; BMI, body mass index; GRWR, graft-recipient weight ratio; HCC, hepatocellular carcinoma; LRT, locoregional treatment; LT, liver transplantation; MELD, model for end-stage liver disease; PIVKA II, protein induced by vitamin K absence or antagonist-II; PVTT, portal vein tumor thrombus.

Both groups had similar AFP (5.2 [2.8–28.6] ng/ml vs. 6.4 [3.2–23.9] ng/ml, *P*=0.707) and PIVKA II (31 [19–60] mAU/ml vs. 29 [19–73] mAU/ml, *P*=0.625) levels at liver transplantation (LT). Pretransplant locoregional therapy (LRT) (81.4% vs. 79.4%, *P*=0.846), systemic treatment (1.7% vs. 2.7%, *P*=0.942), and prior liver resection (8.5% vs. 14.6%, *P*=0.254) were not different between the groups. All explant pathology components were similar between the two groups except for satellite nodules, which were more frequent in the GRWR <0.7 group than in the control group (33.9% vs. 21.4%, *P*=0.034). After PS matching, all characteristics were well-balanced between the GRWR <0.7 (*n*=48) and GRWR ≥0.7 groups (*n*=198), except pretransplant LRT, for which the standardized mean difference was 0.10.

### GRWR less than 0.7 and HCC outcomes

**Figure 2:**
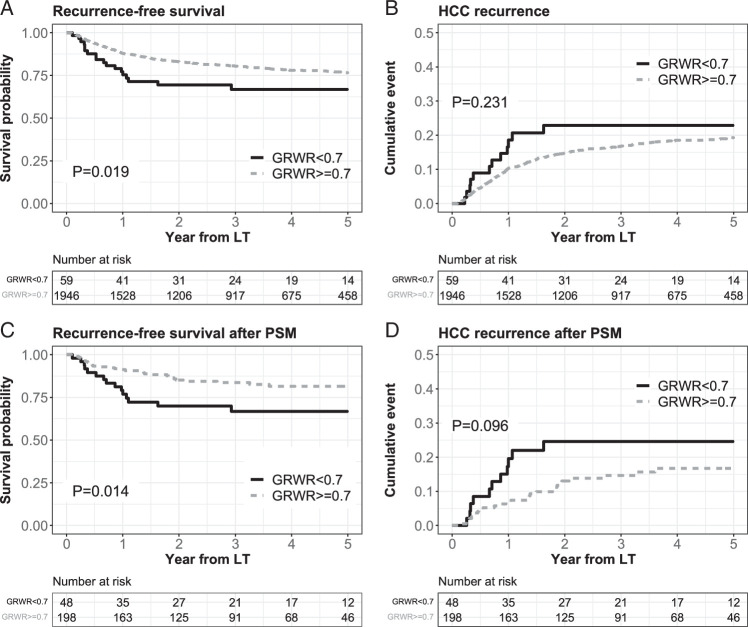
Recurrence-free survival and HCC recurrence before and after matching. GRWR, graft-recipient weight ratio; HCC, hepatocellular carcinoma; LT, liver transplantation; PSM, propensity score matching.

In the entire population, the 5-year RFS was significantly lower in the GRWR <0.7 group than in the GRWR ≥0.7 group (66.7% in the GRWR <0.7 group vs. 76.6% in the GRWR ≥0.7 group, P=0.019, Fig. 2). The 5-year HCC recurrence was not different between the two groups (22.9% vs. 19.3%, *P*=0.231). In the matched population, RFS was also significantly lower in the GRWR <0.7 group than in the GRWR ≥0.7 group (66.9% vs. 81.5%, *P*=0.014). The difference in HCC recurrence did not reach statistical significance after PS matching (75.5% and 83.3%, *P*=0.096). In the multivariable Cox analyses, GRWR <0.7 was an independent risk factor for RFS [adjusted HR (aHR) 1.89, 95% CI 1.15–3.10, *P*=0.012, Table 2], although the result for HCC recurrence was marginal (aHR 1.61, 95% CI 0.97–2.88, *P*=0.066).

**Table 2:** Multivariable Cox analyses for HCC outcomes.

	RFS		HCC recurrence	
Variables^a^	HR (95% CI)	*P*	HR (95% CI)^b^	*P*
GRWR <0.7 vs. ≥0.7	1.89 (1.15–3.10)	0.012	1.61 (0.97–2.68)	0.066
Age			0.99 (0.98–1.01)	0.237
Sex, male	1.43 (1.04–1.97)	0.027	1.66 (1.13–2.44)	0.009
BMI, kg/m^2^	0.95 (0.92–0.98)	0.003	0.95 (0.91–0.98)	0.002
MELD	1.01 (0.99–1.03)	0.276	0.99 (0.97–1.01)	0.422
ABO incompatibility	1.21 (0.97–1.51)	0.093		
Donor age	1.01 (1.00–1.02)	0.062		
Log AFP at LT	1.10 (1.05–1.16)	<0.001	1.12 (1.07–1.18)	<0.001
Log PIVKA II at LT	1.08 (1.02–1.16)	0.014	1.10 (1.02–1.18)	0.009
Pretransplant LRT	1.46 (1.12–1.90)	0.006	1.54 (1.14–2.09)	0.005
Prior liver resection	1.69 (1.30–2.19)	<0.001	1.85 (1.40–2.45)	<0.001
Viable tumor number	1.03 (1.01–1.05)	0.001	1.03 (1.02–1.05)	<0.001
Maximum tumor size, mm	1.12 (1.08–1.17)	<0.001	1.13 (1.08–1.18)	<0.001
Microvascular invasion	2.18 (1.72–2.78)	<0.001	2.61 (2.00–3.41)	<0.001
Satellite nodule	1.01 (0.79–1.28)	0.961	1.35 (1.06–1.73)	0.017
Poor differentiation	1.27 (1.01–1.60)	0.037	1.05 (0.81–1.37)	0.689
Pretransplant systemic treatment	2.75 (1.80–4.19)	<0.001	2.03 (1.19–3.46)	0.010
PVTT	2.11 (1.40–3.17)	<0.001	2.36 (1.56–3.57)	<0.001

a Results were shown with only variables which were included in the multivariable models. Full results are provided as Supplementary Table S1 (Supplemental Digital Content 2, http://links.lww.com/JS9/C491) and Supplementary Table S2 (Supplemental Digital Content 2, http://links.lww.com/JS9/C491).

b Multivariable analysis for HCC recurrence was performed, treating non-HCC death as a competing risk.

AFP, alpha-fetoprotein; BMI, body mass index; GRWR, graft-recipient weight ratio; HCC, hepatocellular carcinoma; LRT, locoregional treatment; LT, liver transplantation; MELD, model for end-stage liver disease; PIVKA II, protein induced by vitamin K absence or antagonist-II; PVTT, portal vein tumor thrombus.

### Subgroup analyses according to tumor burden

In subgroups with lower tumor burden, such as those meeting the Milan criteria or with a MoRAL score in the first through third quartile, RFS was not different regardless of the GRWR (87.1% vs. 85.5, *P*=0.972 in the subgroup meeting the Milan criteria and 80.1% vs. 82.4%, *P*=0.524 in the subgroup with a MoRAL score in the first through third quartile, Supplementary Fig. S3, Supplemental Digital Content 2, http://links.lww.com/JS9/C491). However, when the tumor burden was high (exceeding the Milan criteria or a MoRAL score in the fourth quartile), RFS was significantly lower in the GRWR <0.7 group than in the GRWR ≥0.7 group (18.8% vs. 57.0%, *P*<0.001 in the subgroup exceeding the Milan criteria and 19.2% vs. 59.0%, *P*<0.001 in the subgroup with a MoRAL score in the fourth quartile). Correlations between GRWR <0.7 and RFS in the high tumor burden subgroups were significant in the multivariable Cox models (aHR 3.44, 95% CI 1.89–6.26, *P*<0.001 in the subgroup exceeding the Milan criteria and aHR 3.33, 95% CI 1.69–6.56, *P*<0.001 in the subgroup with a MoRAL score in the fourth quartile).

**Figure 3:**
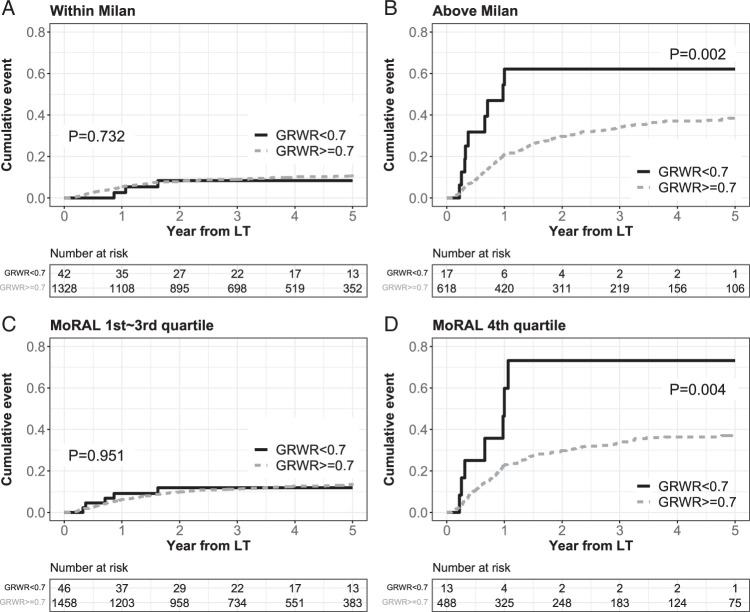
Subgroup comparison of HCC recurrence according to pretransplant tumor burden. The cutoff for the fourth quartile of the MoRAL score was 112.6. GRWR, graft-recipient weight ratio; HCC, hepatocellular carcinoma; LT, liver transplantation; PSM, propensity score matching.

HCC recurrence was also similar regardless of the GRWR when the tumor burden was low (8.4% vs. 10.7%, *P*=0.732 in the subgroup meeting the Milan criteria and 11.9% vs. 13.5%, *P*=0.951 in the subgroup with a MoRAL score in the first through third quartile, Fig. 3). However, HCC recurrence was significantly higher in the GRWR <0.7 group than in the GRWR ≥0.7 group when the tumor burden was high (62.1% vs. 38.4%, *P*<0.001 in the subgroup exceeding the Milan criteria and 73.2% vs. 37.0%, *P*<0.001 in the subgroup with a MoRAL score in the fourth quartile). Correlations between GRWR <0.7 and HCC recurrence were significant in multivariable Cox models in high tumor burden subgroups (aHR 2.77, 95% CI 1.36–5.61, *P*=0.005 in the subgroup exceeding the Milan criteria and aHR 2.61, 95% CI 1.17–5.81, *P*=0.019 in the subgroup with a MoRAL score in the fourth quartile). These correlations with HCC outcomes in subgroups with a high tumor burden were significant for the GRWR <0.7 group but not for the GRWR 0.7–0.8 group (Supplementary Table S3, Supplemental Digital Content 2, http://links.lww.com/JS9/C491).

## Discussion

Amid the ongoing organ shortage, the need for LDLT has been increasing worldwide^26^. LDLT would also be a good curative treatment option for HCC^4^. However, the oncologic risk of small-sized grafts in LDLT is still an important concern that has not been sufficiently investigated in terms of tumor burden and optimal graft size cutoff. This study revealed that a liver graft with a GRWR <0.7 (not <0.8) had the potential to increase HCC recurrence when patients exceeded the Milan criteria or had a high MoRAL score, according to one of the largest LDLT cohorts in the world. Our results could provide good clinical indications for performing LDLT with small-sized grafts when considering the regional deceased donor pool and tumor burden.

Unlike several single-center studies, recent large-cohort studies showed that LDLT had HCC outcomes similar to those of deceased donor liver transplantation (DDLT) when the pretransplant tumor burden was well-controlled, and LDLT even had a survival benefit compared with DDLT according to an intention-to-treat approach^2–4,27^. However, a Korean single-center study showed that RFS in the GRWR <0.8 subgroup was inferior to that in the GRWR ≥0.8 subgroup for LDLT for HCC within a group that exceeded the Milan criteria^17^. That study did not present the difference in time to recurrence, which is an important outcome when investigating the oncologic risk in HCC patients^28^. The lower RFS in the study may not have resulted from HCC recurrence but from the lower survival rate of grafts with a GRWR <0.8^16^. In our study, neither HCC recurrence nor RFS were different, regardless of tumor burden, when the GRWR cutoff was set to 0.8. Using multicentric large-volume data, we demonstrated that a GRWR <0.7 was the cutoff for increased HCC recurrence after LDLT.

Despite the increased oncologic risk, the feasibility of LDLT with grafts with a GRWR <0.7 should be based on comparisons with other patients on the waitlist or other HCC treatments. Additionally, liver function should be taken into account because the severity of cirrhosis hinders most HCC treatments other than LT^29^. In cases in which proceeding with LDLT with a small-sized graft is inevitable in patients with a high HCC burden, aggressive portal flow modulation could be considered to reduce graft injury during reperfusion and systemic inflammation^30^. However, there is no evidence indicating whether portal flow modulation could reduce HCC recurrence.

Further research is needed to determine whether grafts with a GRWR <0.7 increase intrahepatic recurrence or distant metastasis. Extrahepatic metastasis is reportedly more common than intrahepatic recurrence, which accounts for only 15–40% of cases of HCC recurrence after LT^31^. The recurrence site did not differ regardless of donor type and GRWR in previous studies^5,17^. We could not analyze the recurrence site because the KOTRY has only collected these data since 2020. If there is a difference in the recurrence pattern for small-sized grafts, this could be important information for the treatment strategy or adjuvant treatment to reduce tumor recurrence in patients undergoing LDLT for HCC.

In cases of favorable tumor biology and certain recipient circumstances, LT could result in excellent survival in patients with morphologically advanced HCC^32^. Therefore, ongoing efforts are underway to expand LT candidates among patients with HCC, including patients with a portal vein tumor thrombus and even lung metastasis^33,34^. Based on the results from this study, we suggest the following approaches when a GRWR <0.7 is expected during planning for LDLT in patients with a high HCC burden such as those exceeding the Milan criteria or with a high MoRAL score: (1) discuss other eligible living donors, (2) undergo further LRT or systemic treatment to downstage HCC to within the Milan criteria or to achieve a low MoRAL score and then proceed to LDLT with GRWR <0.7 graft if downstaging is successful, (3) wait for DDLT instead of LDLT while undergoing repeated HCC treatment if downstaging fails, (4) proceed to LDLT with a graft with a GRWR <0.7 despite the high tumor burden if the regional deceased donor pool is not sufficient or if deterioration of liver function is sufficiently accelerated to receive urgent LDLT. These strategies should be followed based on comprehensive consideration of tumor aggressiveness, the regional deceased donor pool and allocation policy, and willingness for LDLT of the living donor and recipient.

A lack of imaging data at diagnosis and pretransplantation is a limitation of this study that prevented more precise models for HCC recurrence from being included in the analyses. However, our multicentric data sufficiently showed the oncologic risk of grafts with a GRWR <0.7 using detailed explant pathology. Lack of portal flow modulation, splanchnic hemodynamics, and consequent small-for-size syndrome are other limitations of this study. Further investigation should be performed to determine the effects of these parameters on HCC recurrence in LDLT using small-size grafts.

## Conclusion

Living liver grafts with a GRWR <0.7 resulted in lower RFS and higher HCC recurrence after LDLT than for those with a GRWR ≥0.7 when the tumor burden was high. Adequate strategies are needed regarding the tumor burden, other eligible living donors, and the regional deceased donor pool when the GRWR is expected to be less than 0.7 in LDLT for HCC patients.

## Ethical approval

The institutional review board of Severance Hospital approved the study (4-2023-1550).

## Consent

Patient consent for this study was waived because of its retrospective design.

## Sources of funding

There are no sponsors in this study.

## Author contribution

D.-G.K. and D.J.J. had full access to all aspects of the study and takes responsibility for the integrity of the data and the accuracy of the data analysis. D.-G.K., A.P., K.-H.K., A.S., and D.J.J.: research design; S.H., K.W.L., J.M.K., Y.K.Y., D.C., J.H.R., B.W.K., D.S.K., J.Y.C., Y.W.N., M.K.J., T.-S.K., J.G.L., M.S.K., and S.J.N.C.: performance of the research; D.-G.K., S.J.N.C., and D.J.J.: data acquisition; D.-G.K. and A.P.: statistical analysis; D.-G.K., A.P., and A.S.: writing of the paper; S.J.N.C. and D.J.J.: supervised the study process.

## Conflicts of interest disclosure

The authors have no conflicts of interest.

## Research registration unique identifying number (UIN)

1. Name of the registry: CRIS (Clinical Research Information Service).
2. Unique identifying number or registration ID: KCT009446.
3. Hyperlink to your specific registration (must be publicly accessible and will be checked): https://cris.nih.go.kr/cris/member/my/myCris.do

## Guarantor

Dong Jin Joo, MD, PhD, Department of Surgery, Yonsei University College of Medicine, 50-1 Yonsei-ro, Seodaemun-gu, Seoul 03722, South Korea; Tel.: +82 2 2228 2131; fax: +82 2 313 8289; e-mail: djjoo@yuhs.ac

## Data availability statement

Datasets generated during the current study are available upon reasonable request.

## Provenance and peer review

Not commissioned, externally peer-reviewed.

## Supplementary Material
