# Supplementary material for: Small graft size and hepatocellular carcinoma outcomes in living donor liver transplantation: a retrospective multicentric cohort study
Source: Int J Surg. 2024 May 3;110(8):4859–66. doi: 10.1097/JS9.0000000000001532 (PMC11325899; doi:10.1097/JS9.0000000000001532)
Supplement: Supplementary file 2 [file js9-110-4859-s002.docx]

**Figure S1. Study population**

LDLT, living donor liver transplantation; HCC, hepatocellular carcinoma; KOTRY, Korean Organ Transplant Registry; GRWR, graft-recipient weight ratio

**Figure S2. Distribution of GRWR measured before graft implantation**


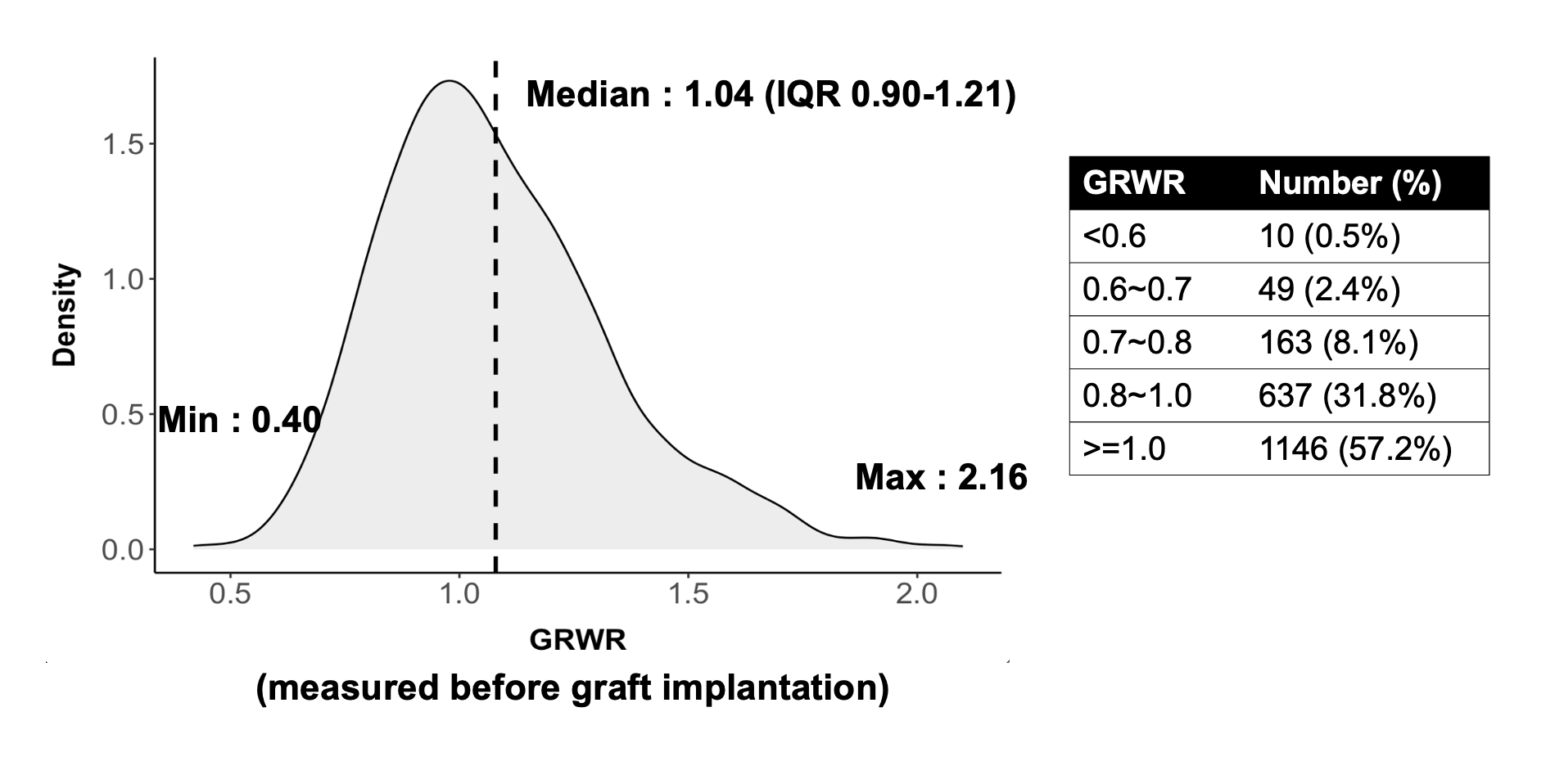


**Table S1. Full result of multivariable Cox regression for RFS**


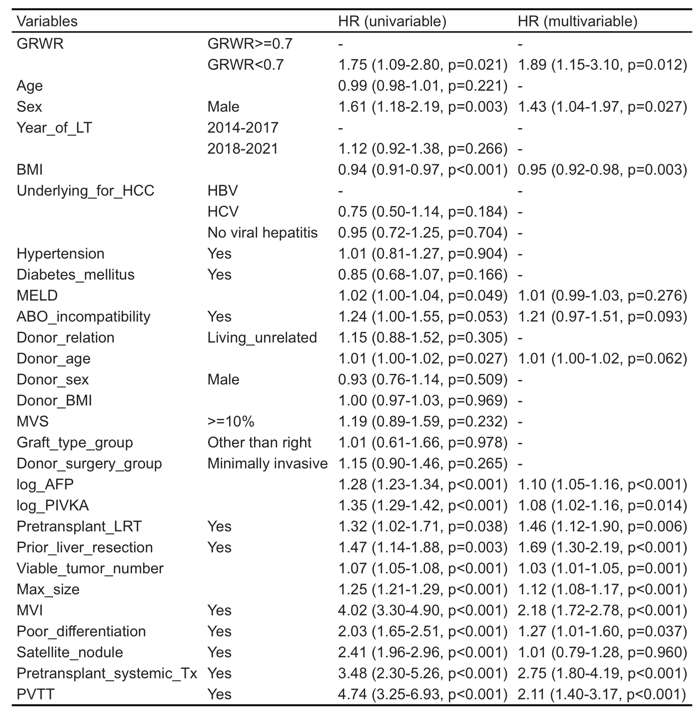


**Table S2. Full result of multivariable Cox regression for HCC recurrence**


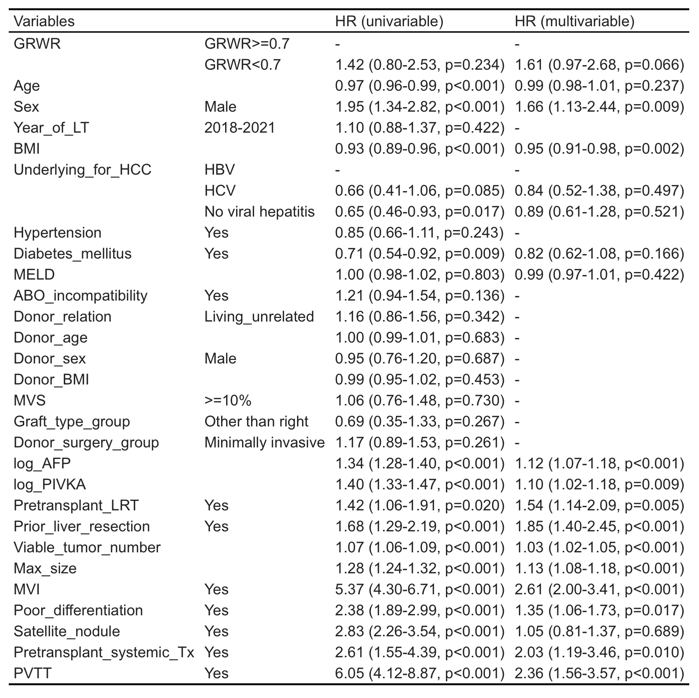


**Figure S3. Subgroup comparison of RFS according to pretransplant tumor burden**

Cutoff for 4^th^ quartile of MoRAL score was 112.6.

HCC, hepatocellular carcinoma; PSM, propensity score matching; LT, liver transplantation; GRWR, graft-recipient weight ratio


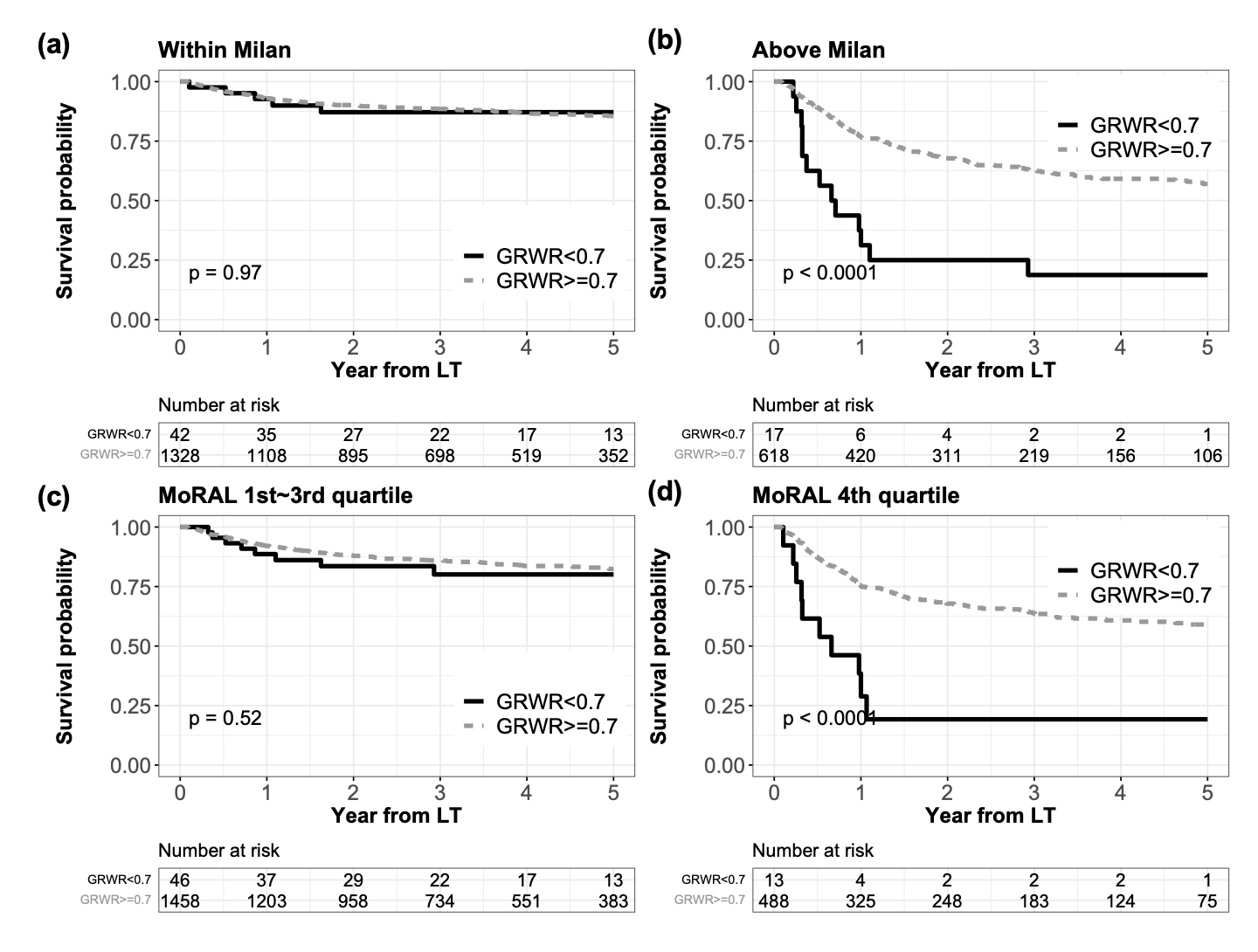


**Table S3. Multivariable Cox analyses for the correlation between GRWR and HCC outcomes according to tumor burden subgroups**
